# Impact of Psychosocial Factors on the Activity of Crohn’s Disease: A Cross-Sectional Analysis of Social Support, Stress, and Flare-Up Incidence

**DOI:** 10.3390/jcm13113086

**Published:** 2024-05-24

**Authors:** María José de Dios-Duarte, Andrés Arias, Ana Barrón

**Affiliations:** 1 Faculty of Nursing, Nursing Department, University of Valladolid, 47005 Valladolid, Spain; 2 Faculty of Social Work, Social Work Department, Complutense University of Madrid, 28223 Madrid, Spain; aariasas@ucm.es; 3 Faculty of Psychology, Social Psychology Department, Complutense University of Madrid, 28223 Madrid, Spain; abarronl@ucm.es

**Keywords:** Crohn’s disease, flare-up, social support, stress

## Abstract

**Background/Objectives**: Crohn’s disease is a chronic and debilitating intestinal disorder that alternates between remission and active flare-ups, often leading to hospitalization. Social support is known to enhance adaptation to the disease and modulate stress perception in patients, while stress may exacerbate symptoms. The aim of this study was to examine the roles of perceived stress and social support in Crohn’s disease and their impact on the frequency of flare-ups. **Methods**: A cross-sectional observational study was conducted, assessing stress and social support in a cohort of 91 patients with Crohn’s disease during flare-up and remission phases. The Perceived Stress Scale (PSS-14) and a Social Support Questionnaire were utilized for evaluation. We examined the relationship between stress and social support in Crohn’s disease. The interaction between the variables studied was also observed, considering the stage of the disease. Finally, we carried out an analysis of the influence of these two variables on the development of flare-ups in Crohn’s disease. **Results**: The study revealed that patients experience higher stress levels during flare-ups and that these levels are amplified by a lack of social support. A significant relationship was identified between the levels of social support and the occurrence of flare-ups, indicating that better social support is associated with fewer flare-ups. **Conclusions**: Patients with Crohn’s disease in the flare-up phase are subject to considerable stress. A deficit in social support is linked to an increase in stress levels. The interaction between social support and stress plays a critical role in the development of flare-ups.

## 1. Introduction

### 1.1. Crohn’s Disease

Crohn’s disease is an inflammatory bowel disease of unknown origin that can affect any section of the gastrointestinal tract, from the mouth to the anus [1,2]. Determining the exact incidence of new cases each year is challenging due to inconsistencies in reporting and variations in diagnostic criteria. Nevertheless, it is recognized as a condition of rising incidence, especially in Western countries [3]. The symptoms commonly include abdominal pain, diarrhea, fatigue, weight loss, and, in more severe cases, the occurrence of fissures, abscesses, or fistulas that may require surgical intervention. Additionally, surgery might be necessary to remove non-functional segments of the bowel. The disease’s clinical course is often punctuated by episodes of remission and flare-ups, which significantly affect the patient’s overall well-being. The progression of the disease is marked by flare-ups, with a relapse risk estimated to be between 30% and 60% per year [4]. Identified as an autoimmune disorder, the immune system in Crohn’s disease mistakenly attacks the body’s own tissues as if they were foreign antigens. It is a chronic, progressive, relapsing, and highly debilitating disease and the primary treatment goal is to manage the signs and symptoms [5]. While the etiology of Crohn’s disease is not fully understood, it is hypothesized by healthcare professionals that stress levels and the presence or absence of social support can influence the likelihood of relapse in individuals with this condition.

### 1.2. Stress

Psychological stress arises from the interaction between an individual and their environment, which they perceive to be threatening or surpassing their resources, potentially compromising their well-being [6]. Stress, therefore, is not inherent to the individual or the environment but rather, is a product of the unique interaction between them. It is also a response to the demands placed upon an individual that may be incompatible with their normal adaptation to the environment, potentially impacting both physical and psychological health [7].

Studies specifically concerning Crohn’s disease have indicated that stress affects the course of the disease, treatment response, and exacerbation of symptoms, including the onset of flare-ups. A study from 2011 [8] highlighted that stress impacts the brain–gut axis, precipitating the release of neurotransmitters and pro-inflammatory cytokines, leading to alterations in gastrointestinal motility and adverse effects on the intestinal mucosa and microbiota. Similarly, research by De Punder and Pruimboom in 2015 [9], confirmed that stress not only exacerbates the disease but also causes inflammation of the intestinal mucosa, increasing permeability and enabling the entry of foreign substances. This effect of stress on the intestinal barrier has been corroborated by a review of various studies conducted in 2019 [10].

In the same vein, research led by Shaler et al. [11] demonstrated that psychological stress contributes to the exacerbation of Crohn’s disease by inducing changes in the gut microbiota, which may play a role in its pathogenic process, influencing protective immunity.

Furthermore, the role of social support has been identified as a significant factor in the context of stress. Professional experience suggests that social support is crucial, not only for its direct impact but also for its indirect influence on stress levels, which, in turn, affect the stress relationship in the exacerbation of Crohn’s disease.

### 1.3. Social Support

Social support is recognized as a pivotal psychosocial factor that influences the perception and development of coping mechanisms, facilitating adaptation to various circumstances [12,13]. It also serves as a mitigating element in stress associated with illness [14,15,16]. Social support includes real support interchanges, the individual’s perception of such doings, and their satisfaction with the assistance provided [14].

Seminal research on the psychological benefits of social support in the context of illness was conducted by Cassel [16]. Another early contributor to this field, Cobb [17], posited that the advantages of social support stem from providing individuals with the certainty that they are cared for and valued within their social networks.

The beneficial impacts of social support have been underscored in numerous studies [18,19,20]. Social support refers to the array of emotional, material, informational, or companionship resources that a person perceives or receives from various members of their social network [14].

### 1.4. Social Support and Stress: Hobfoll’s Model

Hobfoll’s model focuses on investing in or acquiring resources to be used in the coping process [21].

Moreover, the model considers a series of strategies through which social support resources strengthen resistance to stress, both preemptively (preventive or primary) as well as after an individual has been exposed to it (palliative or secondary). Social support provides a sense of being connected and mitigates the perception of loneliness, prevents individuals from experiencing the loss of additional resources, and can directly deter resource loss by averting stress. Once stress is perceived, social support can directly or indirectly replenish lost resources and it can also promote more effective use of available resources by a stressed individual, as well as foster the creation of new ones. The protective role of social support against stress has been corroborated by several studies [22,23]. A study in 2022 [24] showed that those with scant social support exhibit greater activation of the stress circuit (ventral medial prefrontal cortex, dorsal striatum, and periaqueductal gray), which is not observed in individuals with ample social support. Therefore, high levels of social support diminish both the neural and subjective stress responses, whereas low levels of social support intensify the neural stress response.

Regarding Crohn’s disease, specific studies focusing on social support are lacking. Research on other digestive disorders and inflammatory bowel disease at large exists. For instance, Lee and colleagues [25] discovered that patients with functional dyspepsia perceived less social support compared to healthy individuals. In the context of inflammatory bowel disease, Sewitch et al. [26] found that the interaction between stress and disease activity was inversely proportional to satisfaction with perceived social support.

In conclusion, there is scarce research on these two variables and their impact on the onset of flare-ups in Crohn’s disease. Hence, investigating the relationship between stress, social support, and their influence on the occurrence of flare-ups in Crohn’s disease is imperative.

This study aims to examine the roles of perceived stress and social support in Crohn’s disease and their influence on the development of flare-ups, based on the hypothesis that stress impacts the occurrence of Crohn’s disease flare-ups and that social support is implicated in this relationship.

We tried to improve the situation of these patients by intervening in the study variables. Initially, we thought it would be useful to carry out preventive actions on social support and stress, in order to help patients adapt to the disease and reduce the number of hospitalizations for Crohn’s disease flare-ups.

## 2. Materials and Methods

### 2.1. Design

A cross-sectional observational study was carried out, employing validated scales to assess the impact of both stress and social support on Crohn’s disease.

### 2.2. Participants

The study was conducted at the Digestive Unit of the Gregorio Marañón University Hospital, focusing on patients diagnosed with a flare-up of Crohn’s disease, and at the Madrid Association for Crohn’s Disease and Ulcerative Colitis. The hospitalized group experiencing flare-ups was admitted, presenting symptoms such as cachexia, fever, vomiting, and, in some cases, pseudo-obstruction of the bowel. These patients had been previously diagnosed with Crohn’s disease through diagnostic tests such as blood tests, stool analysis, colonoscopy, upper gastrointestinal (GI) series, and computed tomography (CT). All were on daily pharmacological and dietary regimes.

The out-patient group associated with the Madrid Association for Crohn’s Disease and Ulcerative Colitis consisted of individuals in the remission phase of Crohn’s disease. They were asymptomatic during the study and were not being treated with Prednisone. These individuals reported a good quality of life, with no abdominal pain and passing 0 to 2 formed stools per day without rectal bleeding.

To be included in the study, patients had to meet the following criteria:

- Be part of the Gregorio Marañón Hospital care are;
- Be diagnosed with Crohn’s disease by a Digestive Specialist;
- Be diagnosed with Crohn’s disease between the ages of 17 and 40 (Montreal classification—A2);
- Disease located in the terminal ileum or colon or ileocolic (L1 or L2 or L3—Montreal classification);
- Clinical pattern B1—non-stenosing, non-fistulizing or inflammatory, or B2—stenosing—(Montreal classification);
- Patients in the remission phase had to score less than 5 on the Harvey–Bradshaw Index;
- Patients in the flare-up phase had to score in moderate or severe disease on the Harvey–Bradshaw Index.

Exclusion criteria were

- Any chronic organic disease other than Crohn’s disease;
- Having a psychological disorder diagnosed by a Psychiatric Specialist;
- Having a psychiatric disorder diagnosed by a Psychiatric Specialist.

### 2.3. Procedure

In all cases, the study was presented to the participants in person and instructions were given on the criteria for participation in the study. Questionnaires were given to those who showed interest in participating in the study, after checking that they had understood everything correctly. 

In all cases, participation was voluntary and disinterested. All signed the Informed Consent form. The study was approved by the Ethics Committee of the Faculty of Psychology, Complutense University of Madrid (ref. 2018/19-022). The sample was collected between July 2019 and February 2020.

### 2.4. Instruments

Three questionnaires were utilized in this research: a socio-demographic questionnaire, the Perceived Stress Scale (PSS-14), and a Social Support Questionnaire.

#### 2.4.1. Perceived Stress Scale (PSS-14)

The adapted version of the Perceived Stress Scale (PSS-14) [27], originally developed by Cohen, Kamarck, and Mermelstein, was employed in this study. The adaptation was conducted by Carrobles [28]. The PSS-14 assesses the extent to which life situations are perceived as stressful. This is a self-report instrument that assesses perceived levels of stress over the past month. It consists of 14 items with a five-point response format (0 = never, 1 = almost never, 2 = occasionally, 3 = often, and 4 = very often). The total stress score is the sum of the scores assigned to each of the items, for which it is necessary to invert the scores of items 4, 5, 6, 7, 9, 10, and 13 (in the following sense: 0 = 4, 1 = 3, 2 = 2, 3 = 1, and 4 = 0). The higher the score, the higher the level of stress perceived by the respondent. 

#### 2.4.2. Social Support Questionnaire

The Perceived Social Support Questionnaire, developed by Díaz-Veiga [29], was used to measure social support. This scale evaluates the level of satisfaction with the social, emotional, material, and informational support received from family, friends, other people, and health professionals. Each subject’s total perceived social support score is calculated by adding the scores assigned by the subject to each of the dimensions of social support mentioned above. The higher the score, the higher the level of social support perceived by the subject.

### 2.5. Statistical Analysis

The socio-demographic questionnaire variables were analyzed using percentages and measures of central tendency, including mean and standard deviation.

To examine the relationship between stress and social support in Crohn’s disease, patients were divided into groups with high and low social support, based on the level of stress they reported. The median was employed as the measure of central tendency to avoid bias. This analysis also utilized mean and standard deviation.

To further explore the relationship between stress and social support, a specific analysis was conducted to determine how these two variables interacted, considering the participants’ disease status (flare-up or remission phase). Pearson’s linear correlation coefficient was applied in this analysis.

A more in-depth examination of the relationship between the stress and social support variables and their impact on the occurrence of flare-ups was achieved through binary logistic regression analysis, distinguishing between groups with high and low social support. This assessed the effect of stress on predicting flare-ups, factoring in the level of social support (low or high).

The global accuracy of the test was gauged using the Area Under the Receiver Operating Characteristic (AUROC) curve.

The Statistical Package for Social Sciences, Version 23 (SPSS 23, Chicago, IL, USA), was utilized for the descriptive statistics, hypothesis testing, and analyses of internal consistency, reliability, and validity of the instruments used in this research. All tests were conducted with a 95% confidence level, considering a *p*-value below 0.05 as significant.

## 3. Results

The sample consisted of 91 patients with Crohn’s disease, with an average age of 34.60 years. Of these, 44 were male (48.36%), ranging in age from 17 to 50 years, with an average age of 35.87 years, and 47 were female (51.64%), ranging in age from 16 to 51 years, with an average age of 33.41 years.

The sample size was estimated on the basis of the number of patients belonging to the care area of the Gregorio Marañón Hospital who had been seen in the previous year in consultations or admitted to the hospital with a diagnosis of a Crohn’s disease flare-up (300). We used the formula that estimates n in finite populations for the sample. Each participant was counted only once, regardless of their number of visits or hospital admissions. The initial sample collected was 102 participants, of which 11 of them had to be eliminated. 

### 3.1. Relationship between Stress and Social Support in Crohn’s Disease

To further examine the behavior of the variables we were studying, the sample was divided to account for participants with high and low social support. This division was made using the median as it is an unbiased measure of central tendency. The cut-off point was set at 68.50 (Mdn = 68.50).

The Student *t*-test indicated that the differences between the stress averages of subjects who were in the flare-up phase and remission phase in the high social support group were statistically significant (*p* = 0.008). In this case, it is observed that the participants who are in the remission phase have lower stress levels than those who are in the outbreak phase and these differences are statistically significant. The results are presented in Table 1. 

In addition, this table shows that subjects experiencing a flare-up are also highly stressed with similar scores and those with low levels of social support also have high levels of stress.

For the Pearson linear correlation coefficient analysis, the two groups of patients were considered separately (flare-up or remission phase). The results are presented in Table 2.

In the variables correlation analysis for the group of Crohn’s patients in the remission phase, there was a significant negative correlation (r = −0.476; *p* < 0.05) between the social support and stress variables.

### 3.2. Relationship between Social Support and Stress and Their Influence on the Prediction of the Existence of Flare-Ups

The aim was to determine the influence of stress and social support on the likelihood of experiencing flare-ups in Crohn’s disease. Accordingly, we examined the impact of stress on the probability of a flare-up, considering the previously performed classification of patients into high and low social support groups.

To further understand the influence of stress in relation to social support in Crohn’s disease, a binary logistic regression model was created. This model used the presence of flare-ups as a binomial and categorical dependent variable, with two possible outcomes: YES or NO. The model was estimated using the introduction method.

In the logistic regression analysis predicting flare-up likelihood for the high social support group, the main effects model was statistically significant (χ^2^ = 7.798; *p* = 0.005). In contrast, the main effects model for the low social support group was not significant (χ^2^ = 1.275; *p* < 0.259), as shown in Table 3.

The predictive power of the model for the high social support group (significant model) explained approximately 31% of the variance in the dependent variable (existence of flare-up), with Nagelkerke’s R^2^ = 0.313. The results are shown in Table 4. The classification table indicated an accuracy of 63.6% for predicting flare-ups (cut-off point: 0.05).

Further analysis in the high social support group examined the relationship between the independent variable (stress) and the dependent variable (existence of flare-up), with results presented in Table 5.

It was found that stress (with an odds ratio of 1.15) significantly predicts the occurrence of flare-ups, making it a reliable predictive variable.

Consequently, the predictive formula for the probability of a flare-up in the group of Crohn’s patients with high social support is

P(flare-up) = 1/1 + e^(2.841−(0.143×stress))^

**Figure 1:**
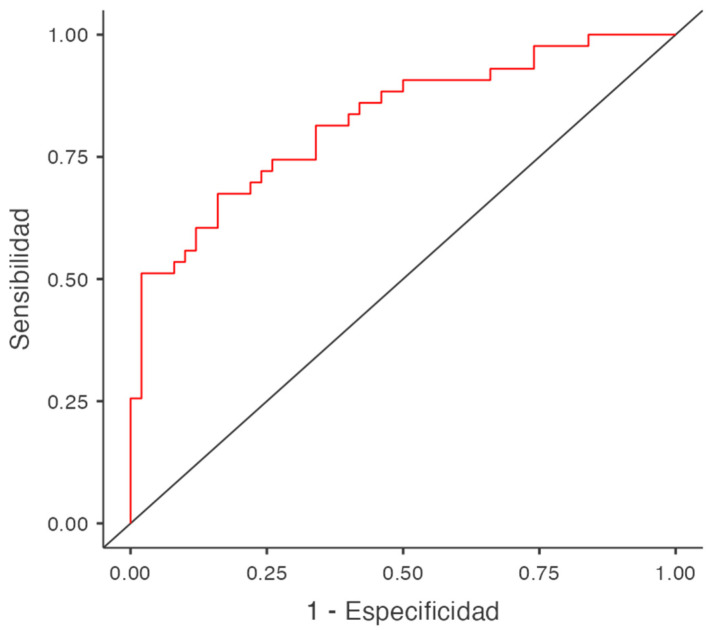
ROC curve for Crohn’s patients belonging to the group with high social support.

Finally, the performance of the statistically significant model found by binary logistic regression in predicting the probability of a flare-up was determined using an ROC curve (see Figure 1). The model has an accuracy of 74.2%, a specificity of 78.0%, and a sensitivity of 69.8%, using a cut-off point of 0.5.

## 4. Discussion

The objectives of this research were to examine the role of perceived stress and social support in Crohn’s disease and their impact on the occurrence of flare-ups.

With regard to stress, the results of our study indicate that people experiencing flare-ups have high levels of stress. These findings are consistent with research in other diseases that demonstrated that stress can trigger disease activity and that increased stress is associated with increased disease symptoms and decreased response to treatment [8,9,10].

Regarding social support, it was found that among participants with high social support, those in the remission phase had lower levels of stress than those in the flare phase (Table 1). These results are statistically significant and indicate that the level of stress is related to the phase the patient is in. Considering previous studies, it could be inferred that the beneficial effect of social support in terms of adjustment to illness and its influence on stress may mediate these findings. Thus, we can conclude from both our results and those found in other research, that social support affects the way patients perceive their disease, in such that it influences the development of coping strategies that allow greater adaptation to the disease, improving the patient’s situation [22,23].

Regarding the impact of social support and stress on the development of flare-ups in Crohn’s disease, we were able to construct a significant predictive model for the likelihood of flare-ups in patients in the high social support group, highlighting the influence of social support on stress levels and the combined effect of these variables on flare-ups. The buffering role of social support on stress (indirect effect) has also been confirmed in studies carried out regarding other diseases [13,14]. 

Consequently, our results indicate that patients require interventions to improve and expand their social support network so that they can benefit from both the direct effect of this variable as well as its buffering effect. We suggest that these patients participate in self-help or support groups. We also propose direct interventions for stress (relaxation techniques, visualization, breathing, meditation, etc.). This will have an impact on the level of stress perceived by these patients by reducing it, thus helping to improve their situation and reduce the devastating consequences it produces. These interventions will have an impact on reducing the likelihood of flare-ups, delaying hospitalization of these patients, improving their management, and, at the same time, improving their quality of life.

The primary strength of this study is the use of mathematical modeling to show the influence of both stress and social support on the development of Crohn’s disease flare-ups.

Another advantage is having demonstrated the direct impact of social support on Crohn’s disease, as well as the indirect effects of social support, which are related to the reduction in patients’ stress levels and their effect on decreasing the likelihood of flare-ups.

Regarding limitations, the most significant is the sample size and type of study. 

We propose future research with larger samples, long-term studies, and the collection of clinical variables such as the number of previous flare-ups and the duration of remission periods. Also, the different types of support that make up the Perceived Social Support Questionnaire, the importance that patients give to these, and their influence on stress and flare-ups in Crohn’s disease, should also be investigated.

## Figures and Tables

**Table 1:** Stress averages according to high social support and low social support groups.

		*n*	Average	*p*-Value
Low social support	Latency	23	23.56	0.262
	Flare-up	26	28.06	
High social support	Latency	16	19.45	0.008 *
	Flare-up	26	28.21	

* Correlation is significant at level 0.01 (bilateral).

**Table 2:** Pearson’s Correlation Matrix considering the groups separately.

		Social Support	Stress
Remission phase	Social support	1	−0.476 *
	Stress	−0.476 *	1
Flare-up	Social support	1	0.057
	Stress	0.057	1

* Correlation is significant at level 0.05 (bilateral).

**Table 3:** Omnibus Test for the coefficients of the model.

SS Grp			Chi-Squared	df	Sig.
Low SS	Step 1	Step	1.275	1	0.259
		Block	1.275	1	0.259
		Model	1.275	1	0.259
High SS	Step 1	Step	7.798	1	0.005
		Block	7.798	1	0.005
		Model	7.798	1	0.005

**Table 4:** Prediction of the probability of a flare-up in subjects with high social support.

	Step	−2 Log of Likelihood	R-Squared Cox and Snell	R-Squared Nagelkerke
High SS	2	31.631 ^(b)^	0.229	0.313

^(b)^ Cut-off point: 0.05.

**Table 5:** Binary logistic regression model for flare-up prediction.

		B	E.T.	Wald	df	Sig.	Exp (B)
High social support	Stress	0.143	0.060	5.707	1	0.017	1.154
	Constant	−2.841	1.422	3.994	1	0.046	0.058

## Data Availability

Data are available upon request through the corresponding author.
